# Toward Economic Growth and Value Creation Through Social Entrepreneurship: Modelling the Mediating Role of Innovation

**DOI:** 10.3389/fpsyg.2022.914700

**Published:** 2022-06-15

**Authors:** Wenjie Wang

**Affiliations:** Chinese Opera, Shandong University of Arts, Jinan, China

**Keywords:** social entrepreneurship, innovation, sustainable economic growth, value creation, SEM technique

## Abstract

The concept of social entrepreneurship emerged as a significant factor that contributes toward public welfare and prosperity. Recent studies showed that social entrepreneurship influences the economic growth and sustainability of the state. Therefore, the underlying aim of this study was to investigate the impact of social entrepreneurship on sustainable economic growth and value creation. This study also undertook to observe the mediating role of innovation in the relationship between social entrepreneurship and sustainable economic growth and between social entrepreneurship and value creation. A questionnaire technique was adopted to obtain data from 343 tour operators in China. The Smart-PLS software was used to analyze the data through the aid of a structural equation modelling (SEM) technique. The results revealed that social entrepreneurship has an effect on sustainable economic growth and value creation. The results also demonstrated that innovation has an effect on sustainable economic growth and value creation. Moreover, it was also observed that innovation mediated the relationship between social entrepreneurship and sustainable economic growth and between social entrepreneurship and value creation. Theoretically, this study made a valuable contribution by examining the impact of social entrepreneurship on sustainable economic growth and value creation and innovation as a mediator. In terms of practical implications, this study would certainly aid the policymakers to devise policies and strategies aim to encourage and promote social entrepreneurship. Moreover, future studies can introduce other mediating and moderating variables in order to gain a deeper insight into the phenomenon.

## Introduction

Recently, the concept of entrepreneurship has been explored and viewed from different perspectives (Ranville and Barros, 2021). One of the ways through which entrepreneurship can be seen is how it affects the economic growth of the country. Higher economic growth leads to a higher rate of employment and better living standards for the people in the society, especially when the economy is down (Doran et al., 2018). This justifies that entrepreneurship is directly related to the economic growth of the country. Thus, policymakers can intervene and add social entrepreneurship as a significant indicator that boosts sustainable economic growth. Moreover, innovation and human capital are also closely linked with social entrepreneurship. Statistical analysis showed that these variables greatly contribute to economic sustainability and growth (Rayamajhee et al., 2022).

Specifically, social entrepreneurship has recently grabbed the attention of researchers and practitioners and this concept has emerged as a prominent factor that contributes to the betterment of society (Kannampuzha and Hockerts, 2019). Businesses have devised policies toward social well-being to mitigate several social issues such as human welfare, poverty, and employment (Lall and Park, 2020). Social entrepreneurship is a significant factor that can boost social change. Additionally, social entrepreneurs work for society without expecting any direct monetary benefit from society in return (Adro and Fernandes, 2021). Moreover, every type of entrepreneurship has some kind of social function, however, social entrepreneurship and traditional entrepreneurship are different in terms of value creation (Stirzaker et al., 2021). Social entrepreneurship creates social value, while, traditional entrepreneurship aims to generate private economic value. Gupta et al. (2020) opined that entrepreneurial venture promotes economic value that is inseparable from social benefits because commercial and social activities are closely related in the real world. Furthermore, in a larger system, opportunity, entrepreneurship, and philanthropy boost economic sustainability and institutional development. Social entrepreneurship is regarded as novel activity and an amalgam of entrepreneurship (developing new ideas) and social cause (working for society; Bozhikin et al., 2019). Extant literature shows that various studies have attempted to examine the phenomenon of social entrepreneurship and its effect on the perspectives of social enterprises, social entrepreneurs, and social ventures (Dwivedi and Weerawardena, 2018).

Important contributions have also been seen in the field of entrepreneurship by the psychologists for providing clear understanding of the behavioral factors that drive the career choices of the entrepreneurs and their success (Gorgievski and Stephan, 2016). As a result, entrepreneurship research has also offered new insights and avenues to the field of behavioral psychology. For example, entrepreneurial practices have set many examples in identifying the different aspects that have characterized the continuous changes in the work domain, i.e., responsibility, uncertainty, flexibility, time pressure, and the insecurity are yet to be addressed with the help of individual proactive behaviors (Mu et al., 2020). Furthermore, the initial stages of a business have observed no or less standards in the daily or routine work roles. This gives the researchers an opportunity to investigate how entrepreneurship shapes the innovation, careers, organizations, and the overall effect on the environment that could affect the growth of the organization. The field of entrepreneurship, social entrepreneurship in particular, shows a high potential for delivering the innovative and novel solutions to the challenges that societies are facing today, for example, climate change and social exclusion (Stephan et al., 2016). Social entrepreneurship is a driving force for innovation (Kickul et al., 2018). Innovation is stemmed from social entrepreneurship because entrepreneurs intend to work on the opportunities that emerged in the market and produce novel products or services (Douglas and Prentice, 2019). In the context of social entrepreneurship, innovation has been highlighted as a significant factor that boosts innovation in society. This study has considered innovation as an essential construct because innovation in the production process is a competitive advantage for firms and societies. This competitive advantage comes when environmental constraints are minimized and economic growth along with technological progress is enhanced (Ho and Yoon, 2022).

Entrepreneurial activities are conducted to solve societal problems and boost sustainable development in the country. Thus, social value is created through innovation, progressiveness, social responsibility, and competitiveness (Adro and Fernandes, 2021). Innovation, in this regard, significantly helps society to grow and achieve sustainability in long term. Moreover, social entrepreneurs are capable of bringing innovation to society to improve the living standards of the people. Innovative activities not only benefit the society of a country but also improve the overall reputation of the state (Doran et al., 2018). Various social causes are regarded as opportunities for social entrepreneurs so that they work on those opportunities and develop new innovative products and services for mitigating those social problems (Crupi et al., 2022).

Sustainable economic development and economic stability are crucial for the country’s growth (Al-Qudah et al., 2022). The social problems such as poverty, food scarcity, unemployment, and human welfare gradually deteriorate the sustainable economic development of the state (Méndez-Picazo et al., 2021). However, social entrepreneurs intervene to mitigate these problems to bring economic prosperity without compromising the ability of future generations (Morales et al., 2021). Sustainable economic development can be brought about through devising favorable and effective economic policies and development strategies. Additionally, scholars argued that there should not be a trade-off between sustainability and economic growth, therefore, studies have been conducted to examine the factors affecting sustainable economic development and how in turn sustainable economic development influence the country’s economy. The political decision-makers of different countries have been devising policies for sustainable economic development (Morris et al., 2020). Sustainable economic stability can be developed through social entrepreneurship as social entrepreneurs work for the betterment of society, which results in better economic conditions (Abad-Segura and González-Zamar, 2021). Furthermore, the increasing interest in societal or social problems faced by economies has led to attention being paid to ways that could mitigate or reduce these issues. For this reason, economic growth has become a prominent debate and the arguments for such debates are built upon sustainable economic development (Aquino et al., 2018).

The Global Entrepreneurship Monitor defined the social entrepreneurial variable as the activities initiatives or organizations that have a certain objective regarding environmental, social, or community aspects (Gupta et al., 2020). These factors cover new ventures that focus on new value creation and social nature (van Lunenburg et al., 2020). Value creation in terms of social entrepreneurship is developed when the resources are combined in a new way for meeting the needs of the society, creating new organizations, and stimulating social change (Douglas and Prentice, 2019). The countries that encourage value creation can create a competitive advantage and have a better and improved reputation. The role of social entrepreneurship in value creation is critical for the economies. Social entrepreneurship is a phenomenon that boost value creation for better living standards of the people. Value creation emerges when the social problems in society are mitigated. Some examples of social problems are poverty, unemployment, homelessness, gender inequality, and unavailability of health care services. Moreover, innovation as a result of social entrepreneurship is also a significant factor that contributes to value creation. Social innovation boosts value creation which in turn improves the living standard of the society. Moreover, the value created by social entrepreneurship is generally in terms of social value. Also, higher sustainability ensures value creation through social entrepreneurship (Bozhikin et al., 2019).

Social entrepreneurship is a novel concept that needs attention as fewer studies have been conducted to understand this concept (Lall and Park, 2020). Al-Qudah et al. (2022) investigated the impact of social entrepreneurship on sustainable economic development and suggested exploring how social entrepreneurship and innovation would influence sustainable economic development. Moreover, the authors also suggested inculcating value creation in the existing model. Moreover, Méndez-Picazo et al. (2021) found that limited studies have been conducted and investigated the factors that boost sustainable economic development. Additionally, lack of evidence present with regard to innovation as a mediator in the context of social development. Therefore, this study aimed to fill the gap in the literature by examining the impact of social entrepreneurship on sustainable economic development and value creation with the mediating role of innovation. Certain objectives have been developed to fill the gap in the social development literature. The objectives of the study are (1) to examine social entrepreneurship on sustainable economic growth, (2) to investigate the role of social entrepreneurship on value creation, (3) to analyze the influence of social entrepreneurship on innovation, (4) to determine the role of innovation on sustainable economic growth, and (5) to examine the effect of innovation on value creation. The objectives to address the mediating role of innovation have also been established and the objectives are (1) to examine the mediating role of innovation in the relationship between social entrepreneurship and sustainable economic growth and (2) to investigate the mediating role of innovation in the relationship between social entrepreneurship and value creation.

This study also developed the research questions that have been answered in the study. The research questions are the following: What is the relationship between social entrepreneurship and sustainable economic development? What is the effect of social entrepreneurship on value creation? What is the influence of social entrepreneurship on innovation? What is the relationship between innovation and sustainable economic growth? and What is the effect of innovation on value creation? The research questions that were also developed to address the mediating role of innovation have also been established and the questions are the following: Does innovation mediate the relationship between social entrepreneurship and sustainable economic growth? and Does innovation mediate the relationship between social entrepreneurship and value creation?

## Review of Literature and Hypotheses Development

This study intends to examine the impact of social entrepreneurship on sustainable economic growth and value creation among tourist operators in China. The study also aimed to analyze the mediating role of innovation between social entrepreneurship and sustainable economic growth and also between social entrepreneurship and value creation. The framework of the study was supported by the theory of social entrepreneurship, which has been explained below.

### Theory of Social Entrepreneurship

Extant literature related to sustainability and social change has used the theory of social entrepreneurship. Explaining the concept of social entrepreneurship requires advanced research on different characteristics and typologies for creating sustainable public wealth rather than focusing on business performance and private wealth. The theory of social entrepreneurship developed by Schumpeter in 1943 focuses on economic growth and highlights the importance of social entrepreneurship for social development *via* viable models and economic sustainability (Yahchouchy and Dzenopoljac, 2022b). According to this theory, social change, social transformation, and social impact are brought by social entrepreneurs. This theory also explains that social entrepreneurship is a significant factor that boosts economic development, and it also plays a vital role in bringing innovation to the country. This study focuses on social entrepreneurship and its impact on innovation, sustainable economic growth, and value creation. Based on the social entrepreneurship theory, social entrepreneurship significantly impacts sustainable growth and innovation for bringing about social change and prosperity for society. This suggests that social entrepreneurship influences public welfare to improve the living standards of the citizens.

A substantial number of studies employed the theory of planned behavior (Aquino et al., 2018) as a framework to understand entrepreneurial career choice (e.g., Moriano et al., 2012), sometimes in combination with personality traits and identity theory (e.g., Obschonka et al., 2012). Other studies sought to understand specific motives, competencies, and career attitudes including attitudes toward the boundaryless career as antecedents of entrepreneurial career choice (Bozhikin et al., 2019). Several studies investigated a broader range of outcomes such as the development of an entrepreneurial identity, entrepreneurial competencies, reemployment, and vocational rehabilitation success (e.g., Alverson and Yamamoto, 2014, Hodzic et al., 2015).

The fourth largest research area concerns cognition and behavior, focusing on the role of mental processes in entrepreneurial decisions and actions. In line with the cognitive psychology tradition that this area builds upon, research in this area includes a substantial number of experiments and vignette studies (in our review, 27%). Such studies aimed to unravel behavioral processes, such as investigating the role of active information seeking, entrepreneurial experience, and divergent thinking in the process of opportunity identification (Méndez-Picazo et al., 2021). Studies also linked specific cognition-related personality characteristics to behavioral outcomes.

### Relationship Between Social Entrepreneurship and Sustainable Economic Growth

Social entrepreneurship and sustainable economic growth together benefit society and assure future development and prosperity (Burić and Moè, 2020). However, the concept of social entrepreneurship and its impact on sustainable economic development are relatively new, and in the last decade, only a few studies showed the relationship between social entrepreneurship and sustainable economic growth (Moriano et al., 2012; Obschonka et al., 2012; Alverson and Yamamoto, 2014). Palacios-Marqués et al. (2019) opined that social entrepreneurial activities have been devised to reduce social problems, as a result, it boosts sustainable economic growth. Sustainable economic growth can be enhanced by competitiveness, social responsibility, boosting social value, and social entrepreneurship (Littlewood and Holt, 2018). Social entrepreneurship greatly contributes to the sustainable development of the organization and also helps the organization to the sustainable development of the society, market, national, regional, and global that require sustainable innovations (Agarwal et al., 2020). In this regard, it becomes necessary to analyze the role of social entrepreneurship to implement sustainable economic growth for society. Moreover, social entrepreneurs are capable of bringing sustainability by innovating and finding new opportunities that emerge in the marketplace. Thus, social entrepreneurship and sustainability work together for the betterment of society.

Sustainable economic growth is inspired by social entrepreneurship (Al-Qudah et al., 2022). Entrepreneurial activities tend to generate wealth, which in turn expands the market leading to high income, new market dynamics, and opportunities (Gupta et al., 2020). This is a traditional type of entrepreneurship in which the main focus is the maximization of wealth. However, social entrepreneurial activities tend to bring positive change into society and work toward economic development. Based on this phenomenon, social entrepreneurship is closely linked to economic growth (Javed et al., 2019). Among the few studies that have been carried out to examine the association between social entrepreneurship and sustainable economic development (Bosma et al., 2018), most of them found a significant association between these constructs (Méndez-Picazo et al., 2021). For example, Al-Qudah et al. (2022) examined the role of social entrepreneurship on economic development from the viewpoint of economic growth. The study found that social entrepreneurship is positively associated with sustainable development. Another recent study by Méndez-Picazo et al. (2021) investigated the impact of social entrepreneurship on economic sustainability from the perspective of environmental sustainability. The study revealed that social entrepreneurship has an influence on economic sustainability through environmental sustainability. There is still room to investigate the relationship between these variables, therefore the following hypothesis has been formulated:

*H1*: Social entrepreneurship has an effect on sustainable economic development.

### Relationship Between Social Entrepreneurship and Value Creation

Entrepreneurship deals with creating new businesses by taking advantage of the opportunities that emerge in the market (Kickul et al., 2018). This phenomenon ensures the higher growth of the organization by bringing sustainability and creating value for the people. In addition to this, social entrepreneurs also ensure value creation by developing new products for the people and working for a social cause (Crupi et al., 2022). It is significant to note that social entrepreneurial initiatives help to serve society and minimize the adverse effects of social problems and issues; therefore, such activities lead toward value creation. Moreover, social entrepreneurs work for society without expecting any personal gains or benefits, and thus, it enhances value creation for the people. By definition, social entrepreneurship is a process of developing solutions to mitigate social problems. Therefore, social entrepreneurship is a phenomenon that boosts value creation because it aims to bring positive change to society and people (Brambilla et al., 2021).

A lack of evidence exists with regard to social entrepreneurship and its impact on value creation. Few studies have shown a positive relationship between these constructs. For instance, Chandra and Paras (2020) analyzed the impact of social entrepreneurship and value creation. The study discussed that social entrepreneurs develop opportunities and work on those opportunities to create value for the public. Recently, Brambilla et al. (2021) determined how social entrepreneurship is linked with social value creation. The result of the study showed that social entrepreneurship is associated with social value creation through sustainable development. This signifies that social value is created by the efforts of social entrepreneurs and how they work toward the improvement of society. However, due to limited studies conducted in this regard, it becomes significant to test the relationship between social entrepreneurship and value creation. Thus, the following hypothesis has been developed to examine the relationship between these constructs:

*H2*: Social entrepreneurship has an effect on value creation.

### Relationship Between Social Entrepreneurship and Innovation

Entrepreneurs, in general, are regarded as innovators as they seek opportunities in the market and develop new innovative products or services (Singh and Gaur, 2018). In the context of social entrepreneurship, this process also brings innovation for the welfare of society (Ahlstrom et al., 2018). This signifies that social entrepreneurship is closely linked with innovation. Moreover, the public sector has greatly recognized social entrepreneurship as a significant process for development and innovation (Kickul et al., 2018). According to van der Have and Rubalcaba (2016), the public shows interest in social entrepreneurship by providing funds and scholars report social entrepreneurship as a critical factor that leads toward economic development. This increased interest in social entrepreneurship and social innovation has allowed the practitioners to devise policies to boost social entrepreneurship in the organization (Douglas and Prentice, 2019). Social entrepreneurs, being change agents, harness innovation for the welfare of the public and to bring change in social equilibrium. Social innovation is a research domain that is closely linked with social entrepreneurship because social actors are seeking new ways for boosting social and political dimensions in the economy.

Crupi et al. (2022) provided theoretical insights related to how innovation arises in the process of social entrepreneurship. Social entrepreneurial activities are carried out for increasing the level of innovation in society to improve the overall living standard of the people (Fridhi, 2021). Studies have been conducted that explained how social entrepreneurship is related to innovation. For example, innovation as self-organization is increased by social entrepreneurship provided the presence of good governance (Ho and Yoon, 2022). Another recent study found that social entrepreneurship not only influences innovation but also a social network, performance, and sustainability (Kickul et al., 2018). Social entrepreneurship has already gained momentum in the business and social paradigm where innovation and technology are dominant factors of the industry. This indicates that innovation has a close association with social entrepreneurship, such as social entrepreneurial activities bringing innovation to the business and the society (van der Have and Rubalcaba, 2016). By definition, social entrepreneurship has three main characteristics, i.e., accountability, social innovation, and sustainability. Hence, social entrepreneurs create social value by working on the arising opportunities through scarce resources and innovative solutions (Singh and Gaur, 2018). Although few studies like these have shown the association between social entrepreneurship and innovation, however, there is a lack of enough evidence that explicitly explains the relationship between these two variables. In order to examine this relation, the following hypothesis has been developed:

*H3*: Social entrepreneurship has an effect on innovation.

### Relationship Between Innovation and Sustainable Economic Growth

Studies have argued that policies related to sustainable economic growth accelerate through innovation; as a result, a win-win situation is created for the public and organizations (Hao et al., 2021). Regarding the influence of innovation on economic growth, the policies for the countries are devised to promote innovation so that sustainability can be enhanced. The existing literature also confirms that sustainable economic growth is a complex phenomenon; however, innovation is a critical factor that can accelerate this process (Dauda et al., 2019). Moreover, countries are devising strategies to enhance sustainable economic growth for achieving competitive advantage. Based on this, recently developed and developing countries are working on bringing innovation within resource-constrained environments (Kickul et al., 2018). In the socio-economic context, sustainable economic growth is crucial for the country’s reputation, and scholars have emphasized that innovation is a factor that can help to boost this growth. According to the previous literature, the three factors that motivate economic growth are innovations, institutions, and entrepreneurship. These aspects are significant for the decision-makers to design procedures to induce innovation (Al-Qudah et al., 2022).

Both social entrepreneurship and innovation play an important role in boosting economic growth and development in the country (Visvizi et al., 2018). Organizations that value innovation and promote creative ideas can achieve sustainable economic growth because innovation is a key to accomplishing the goals and objectives (Macke et al., 2018). Moreover, the innovative behavior of the entrepreneurs enables them to effectively create novel products, which results in high sustainable economic growth (Doran et al., 2018). In this regard, Sarkar and Pansera (2017) selected the energy sector of India to examine the relationship between innovation and sustainable development. The study explicitly showed that entrepreneurship acts as a catalyst between innovation and sustainable development. Morales et al. (2021) also argued that social entrepreneurship and innovation are key drivers of sustainable economic development. Moreover, Huo et al. (2022) examined the role of green innovation on sustainable economic growth through sustainable resource management. This study highlighted that green innovation strongly and positively impacts sustainable economic growth through the mediation of sustainable resource management. Sustainable processes are developed with the help of innovative activities because innovation can lead to higher productivity, inducing higher sustainable economic growth (Adro and Fernandes, 2021).

Innovation has a positive and significant relationship with economic sustainability. Innovation and sustainability together help to boost economic, environment, and social development (Kuzma et al., 2020). Sustainability can be achieved through innovative initiatives taken by the state or the organization (Kannampuzha and Hockerts, 2019). Innovative procedures are mainly developed to use minimum natural resources and boost productivity growth. These procedures significantly impact sustainable economic growth for the organization. Society and organizations want innovation for sustainable growth. Moreover, innovation is recognized as the main source of economic growth, which also greatly contributes to environmental and social stability (Sarkar and Pansera, 2017). This points out the fact that innovation and sustainable economic growth are positively associated with each other. Visvizi et al. (2018) also argued that innovation is a key driver of social inclusive economic growth for sustainability in ICT-enabled solutions. This study also discussed the importance of technological innovations for bringing sustainability because innovation is one of the aspects that would benefit in the future. Studies have been carried out to examine the role of innovation and sustainable economic growth in different contexts. However, there is still room to examine the direct association of innovation with sustainable economic growth. It would be interesting to find that how economic growth accelerates with the help of innovation. In this regard, the following hypothesis has been posited:

*H4*: Innovation has an effect on sustainable economic growth.

### Relationship Between Innovation and Value Creation

Value creation is a process that is closely associated with innovation and innovative activities (Rayamajhee et al., 2022). Innovation not only optimizes the value creation of the single entity but also grabs the attention of the people. Andreassen et al. (2018) proposed innovation as a key indicator of both value acquisition and value creation. Recently, Adro and Fernandes (2021) asserted that value creation originates from complementarity, novelty, and efficiency, and these three aspects are deeply rooted in innovation. Additionally, value creation accelerates through innovation because new products and services are developed by innovative initiatives (Dwivedi and Weerawardena, 2018). Moreover, Lall and Park (2020) studied the role of innovation from the perspective of value creation for societies. The results depicted that value creation of the core of innovation for societies. The authors concluded that innovation positively impacts value creation in terms of technology, imitation, knowledge, and users. Zhang et al. (2021) argued that technological innovation is a significant approach that fosters value creation. However, the authors added that there are other ways to obtain value.

Innovation from the social economics perspective is a complex phenomenon because innovation requires time and effort. Nonetheless, innovation for the whole society creates value creation to a greater degree. For example, Bacq and Aguilera (2022) investigated the phenomenon of innovation using the value creation theory. The study discussed that innovation is a significant factor that influences value creation. Also, the theory explains innovation as a key determinant in the value creation process. This study was conducted from an organizational perceptive. It is of great importance to understand the impact of innovation on value creation in the context of social economics. Also, a lack of evidence is present that explain value creation through innovation. Therefore, this study intended to investigate the impact of innovation on value creation. Thus, the following hypothesis has been proposed:

*H5*: Innovation has an effect on value creation.

### Mediating Role of Innovation

Based on various studies innovation is related to social entrepreneurship and sustainable economic growth. For instance, social entrepreneurs are agents that foster innovation through their entrepreneurial behavior (Lall and Park, 2020). Consequently, innovation is a process that is most likely to accelerate sustainable economic growth and public welfare (Bozhikin et al., 2019). This evidence shows that innovation could be a strong mediator between these variables. Moreover, innovation is a critical indicator that impacts sustainable economic growth for improving living standards and social entrepreneurs are responsible for bringing this innovation (Douglas and Prentice, 2019). Innovations include more competitive production processes by creating new and improved products for the people. In the context of social economics, social entrepreneurship is a process that brings innovation to society and fosters sustainable economic growth (Dar et al., 2022). This study again suggests that social entrepreneurship is a significant aspect that helps in sustainable economic growth *via* innovation.

Social entrepreneurship and sustainable development mainly focus on the quality of life by reconciling the sustainability aspects with social factors. Additionally, social entrepreneurship and innovation have been regarded as key indicators that fuel sustainable economic growth (Eniola, 2020). In this context, it is significant to bring into discussion the aspects of social entrepreneurship and innovations in the progress of sustainable economic development. It becomes important to note that innovation could be a factor to explain the relationship between social entrepreneurship and sustainable development. In this regard, Doran et al. (2018) found that social entrepreneurship has a positive impact on economic growth. The reason behind this positive relationship is the entrepreneurial activities, for example, the development of new products, the introduction of innovation, and opportunities in the market (Méndez-Picazo et al., 2021). The literature has focused on the entrepreneurial activities that stimulate economic growth and devising economic policies for bringing harmony to society. Social entrepreneurial activities are conducted for the prosperity of society and stimulating value creation. According to Méndez-Picazo et al. (2021), the development of new products, innovation, and search for new opportunities positively impact economic growth that consequently influences value creation. This suggests that innovation is a catalyst that enables social entrepreneurship to stimulate value creation. Moreover, social entrepreneurship is positively associated with sustainable development through entrepreneurial activities and facilitating value creation. Thus, it increases the demand of the economy to accelerate sustainable economic growth (Morales et al., 2021). Social entrepreneurs seek emerging opportunities and try to solve environmental and social problems to maximize value creation in society. When society has fewer issues, the value creation fostered by entrepreneurial activities enhances. Kuzma et al. (2020) also argued that the process of social entrepreneurship involves actions and opportunities that try to overcome environmental and social issues by the search for innovative and creative solutions.

**Figure 1:**
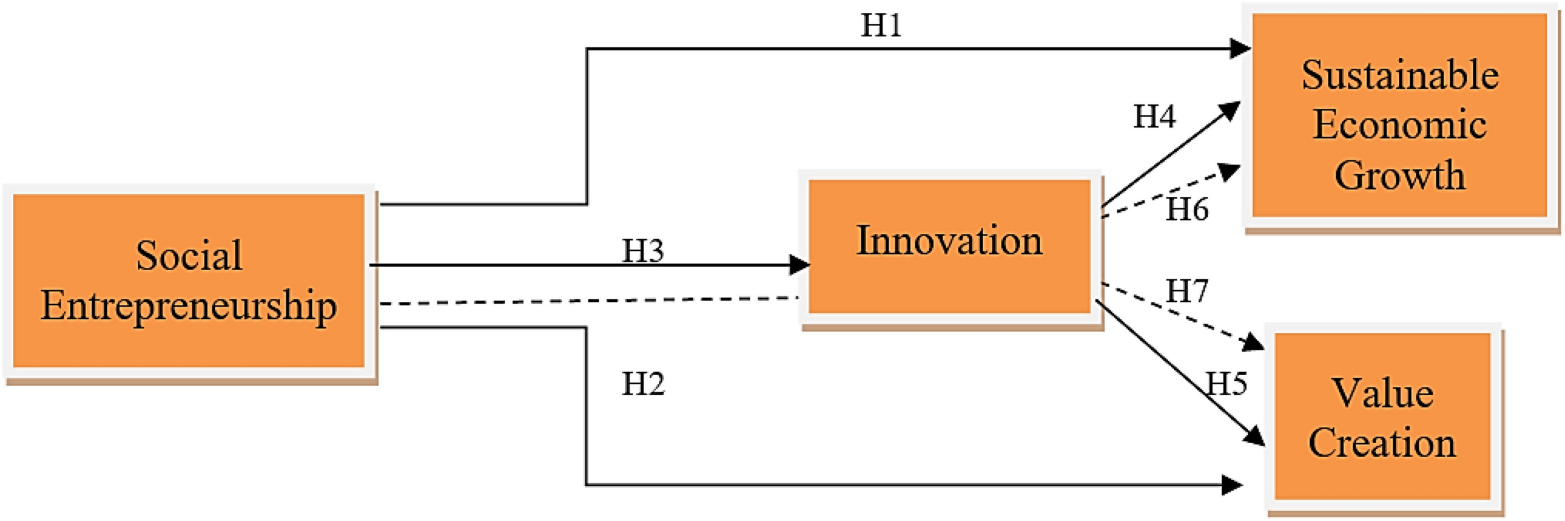
Theoretical framework. SE, social entrepreneurship; inno, innovation; SEG, sustainable economic growth; VC, value creation.

The mediating role of innovation has been studied from different perspectives. For example, Calic and Ghasemaghaei and Turel (2021) examine the mediating effect of innovation in the relationship between big data and corporate social performance. The results showed that innovation mediated the relationship between these variables. Another study conducted by Osei and Zhuang (2020) found that entrepreneurship and social innovation are significant mediators between rural poverty and social capital. Such studies explicitly show that innovation could be a powerful mediator between two positive constructs. Although the mediating effect of innovation has been explored in different contexts, limited studies have analyzed innovation as a mediator in the context of social economics and sustainability. Therefore, to address this gap in the literature, this study opted to examine the mediating role of innovation between social entrepreneurship and sustainable economic growth and between social entrepreneurship and value creation. Thus, the following hypothesis has been formulated. The conceptual framework that was formulated based on the theories and literature is given in Figure 1.

*H6*: Innovation has a mediating role between the relationship of social entrepreneurship and sustainable economic growth.

*H7*: Innovation has a mediating role between the relationship of social entrepreneurship and value creation.

## Methodology

This section presents the methodology that was adopted to examine and investigate the effect of social entrepreneurship on sustainable economic growth and value creation. Moreover, the mediating role of innovation was also studied. This study adopted a quantitative design and a deductive approach to analyze and examine the proposed hypotheses. These hypotheses were formulated to aid the researcher to examine the effect of the predictor variables on the outcome variables. The adoption of a quantitative design aided in eliminating the biases present within the study, so that the reliability of the results may be ensured (Avotra et al., 2021). The process of data collection was carried out with the help of a self-administered survey. To maintain the rationality of the data, the questionnaire was developed in a precise and clear manner. Moreover, the respondents were communicated that there were no right or wrong answers. The respondents were provided the opportunity to be as natural as possible. A total of 400 questionnaires were administered to the participants. The respondents were reminded to fill out the survey forms and return them in a timely manner.

The process of data collection was completed in 2 weeks and a total of 343 forms were obtained. After a thorough screening of the survey forms, 57 forms were discarded as they were either incomplete or improperly filled. Hence, the overall usable response rate was 86%. The data from the usable survey forms were later examined through the aid of statistical software. The target population of this study was comprised of tour operators located in various regions of China. A non-probabilistic convenience sampling approach was used to draw a sample from the population. The adoption of a convenience sampling technique significantly facilitated the researcher to obtain data from readily available respondents in a timely and cost-effective manner (Nawaz et al., 2020; Yingfei et al., 2021). The unit of analysis of this study was individual, and it was comprised of the individual tour operators working in various regions of China.

### Statistical Tool

This study adopted the structured equation modelling (SEM) technique to analyze the data that were obtained from the respondents. For this purpose, the Smart-PLS 3.3.3 statistical tool was used. Hair et al. (2014) posit that the Smart-PLS software helps to conduct a detailed analysis of the data by developing a path model within a short period. This software uses the measurement model (outer model) and the structural model (inner model) to analyze the data (Xiaolong et al., 2021). The validity and reliability of the data are checked through the measurement model, whereas the validities of the proposed hypotheses are confirmed through the structural model. The hypotheses are accepted or rejected based on the t-statistic and *p*-values (An et al., 2021; Nawaz et al., 2022).

### Measurement

The data for this study were gathered through the aid of a five-point Likert scale (1 = strongly disagree, 2 = disagree, 3 = neutral, 4 = agree, and 5 = strongly agree). A detailed description of the measurement scales is given below. There were eight items in the measurement scale of social entrepreneurship, and it was adopted from Naderi et al. (2019). The measurement scale of sustainable economic growth was adopted from Syapsan (2019), and it consisted of three items. There were four items on the scale of value creation, and it was adopted from Naderi et al. (2019). The scale of innovation was comprised of eight items, and it was adopted from Alegre et al. (2009).

### Demographic Profile

The demographic profile of the respondents of the study can be viewed in Table 1. There were 248 males and 95 females who took part in this study. Both the males and females contributed 72.30 and 27.5% to the sample size. Moreover, 65 respondents were aged between 20 and 30 years, 129 were aged between 31 and 40 years, 62 were aged between 41 and 50 years, and 87 were above 50 years of age. A majority of the respondents were aged between 31 and 40 years, and they constituted 37.61% of the total sample. Furthermore, 138 participants had a Bachelor’s education, 149 were holders of a Master’s degree, and 56 participants possessed a Ph.D. or some other educational qualification.

**Table 1:** Demographics analysis.

Demographics	Frequency	Percentage (%)
**Gender**		
Male	248	72.30
Female	95	27.5
**Age (years)**		
20–30	65	18.95
31–40	129	37.61
41–50	62	18.08
Above 50	87	25.36
**Education**		
Bachelor’s	138	40.23
Master’s	149	43.44
Ph.D. and others	56	16.393

*N* = 343.

## Data Analysis and Results

### Measurement Model

**Figure 2:**
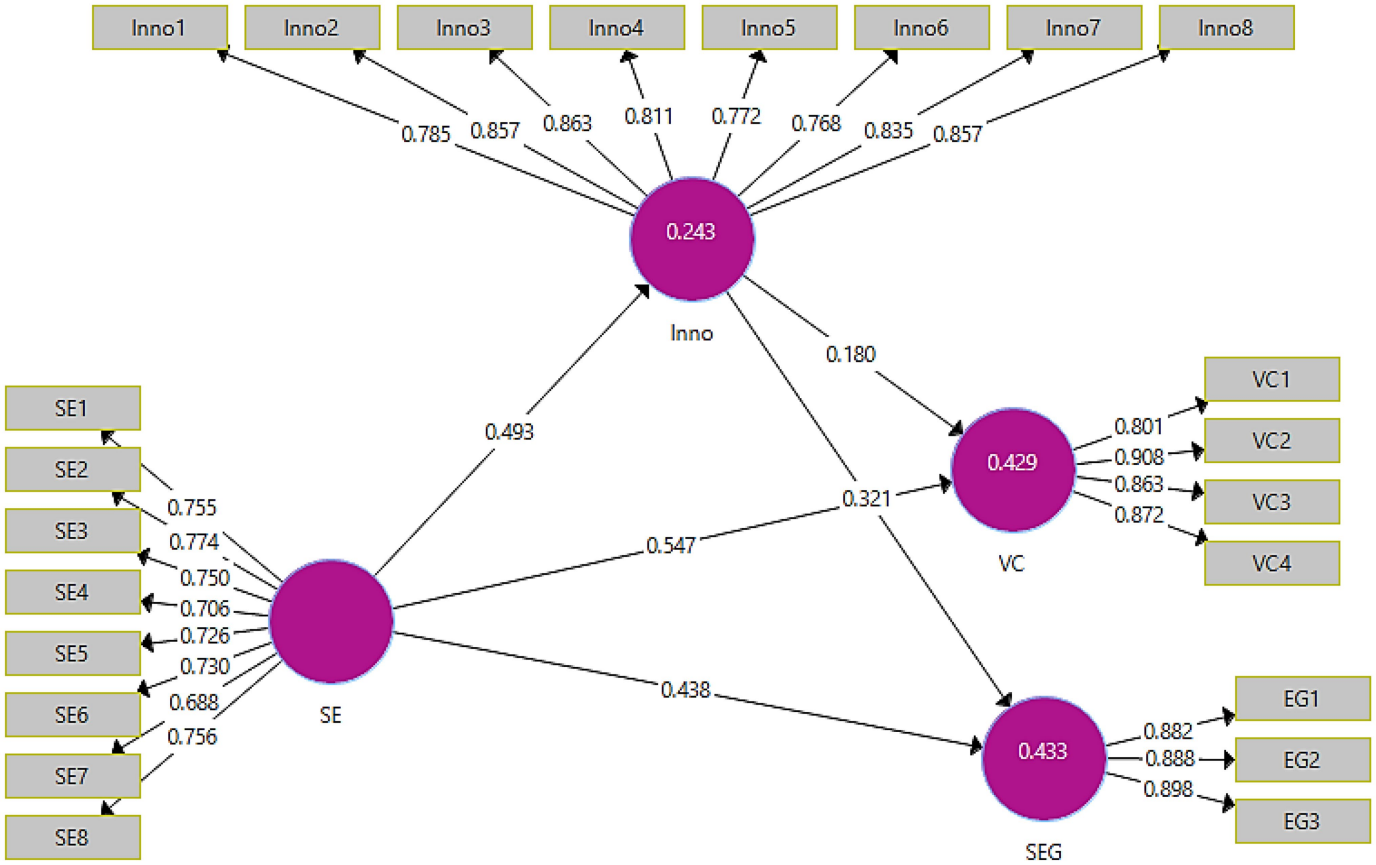
Output of measurement model. SE, social entrepreneurship; inno, innovation; SEG, sustainable economic growth; VC, value creation.

The output of the measurement model can be seen in Figure 2. The assessment of the measurement model demonstrates the extent to which the predictor variables have contributed to the outcome variables of the study.

The assessment of the direct model can be viewed in Table 2. The table demonstrates the values of factor loadings, variance inflation factor (VIF), Cronbach’s alpha, composite reliability, and average variance extracted (AVE) that were obtained against the constructs of the study. Jordan and Spiess (2019) suggest that the desirable factor loadings of the items of the constructs should be above 0.60. It can be viewed that all factor loading successfully met this assumption. The issue of collinearity was addressed using the VIF indicator. Hair et al. (2014) posit that the desirable VIF values should be below 5. It can be viewed that all VIF values ranged between 1.614 and 4.836. Hence, it was ascertained that collinearity did not exist within the proposed model. Furthermore, the construct reliabilities and validities were also checked through the values of Cronbach’s alpha, composite reliability, and AVE. The desirable Cronbach’s alpha value should be above 0.70 (Hair et al., 2017). The alpha values recorded against social entrepreneurship, innovation, sustainable economic growth, and value creation were 0.883, 0.931, 0.868, and 0.884, respectively.

**Table 2:** Model assessment (direct model).

				Construct reliability and validity		
	Factor loadings		VIF	*α*	Composite reliability	AVE
Social entrepreneurship	SE1	0.755	1.975	0.883	0.904	0.542
	SE2	0.774	2.930			
	SE3	0.750	2.530			
	SE4	0.706	1.614			
	SE5	0.726	2.697			
	SE6	0.730	2.605			
	SE7	0.688	2.432			
	SE8	0.756	3.214			
Innovation	Inno1	0.785	3.325	0.931	0.942	0.672
	Inno2	0.857	4.161			
	Inno3	0.863	4.330			
	Inno4	0.811	3.629			
	Inno5	0.772	2.152			
	Inno6	0.768	2.241			
	Inno7	0.835	4.239			
	Inno8	0.857	4.836			
Sustainable economic growth	SEG1	0.882	2.249	0.868	0.919	0.790
	SEG2	0.888	2.254			
	SEG3	0.898	2.287			
Value creation	VC1	0.801	1.666	0.884	0.920	0.743
	VC2	0.908	3.444			
	VC3	0.863	2.601			

VIF, variance inflation factor; α, Cronbach’s alpha; AVE, average variance extracted.

The composite reliability values higher than 0.70 are considered to be satisfactory (Nawaz et al., 2019). It can be viewed from Table 2 that all values of composite reliability were above 0.90. Hence, it was ascertained that the data were reliable. In addition to this, the presence of convergent validity was also confirmed through the values of AVE. The desirable values of AVE should be above 0.50 (Dash and Paul, 2021). The table depicts that all AVE values were higher than 0.50. Therefore, the presence of convergent validity was successfully established.

Table 3 depicts the heterotrait-monotrait (HTMT) ratio and the Fornell and Larcker criterion that was used to confirm the presence of discriminant validity. Discriminant helps in understanding the extent to which a particular variable is unique from the other. Mir et al. (2021) posits that the desirable values of HTMT should be below 0.90. This assumption was successfully met as all HTMT values ranged between 0.464 and 0.710. As far as the Fornell and Larcker criterion is concerned, the general assumption is that the values at the top of each column must be higher than those below them (Henseler et al., 2015). Therefore, based on these results, it can be concluded that discriminant validity existed within the proposed model.

**Table 3:** Discriminant validity.

Fornell–Larcker criterion					Heterotrait–Monotrait ratio				
Constructs	Inno	SE	SEG	VC	Constructs	Inno	SE	SEG	VC
Inno	0.820				Inno				
SE	0.493	0.736			SE	0.500			
SEG	0.537	0.596	0.889		SEG	0.565	0.647		
VC	0.450	0.636	0.625	0.862	VC	0.464	0.684	0.710	

*N* = 343. SE, social entrepreneurship; inno, innovation; SEG, sustainable economic growth; VC, value creation.

The R-square and Q-square values can be viewed in Table 4. These values were recorded against the constructs of the study, i.e., social entrepreneurship, innovation, sustainable economic growth, and value creation. The sustainability of the model was checked through the R-square values, whereas the Q-square values confirmed the predictive relevance of the model. The R-square values should lie close to 0.50. Table 4 shows that all R-square values were close to 0.50. Hence, it can be established that the model was sustainable. Moreover, the desirable Q-square values should be above 0. This assumption was also fulfilled, and therefore, it can be concluded that the model had significant predictive relevance.

**Table 4:** R-square values for the variables.

	R-square	Q-square
Inno	0.241	0.137
SEG	0.430	0.315
VC	0.426	0.288

*N* = 343. SE, social entrepreneurship; inno, innovation; SEG, sustainable economic growth; VC, value creation.

Table 5 presents the collinearity statistics. The issue of collinearity was assessed by observing the inner VIF values. As per Sarstedt et al. (2014), the inner VIF values should be lower than 5. It can be viewed from the table that all inner VIF values were ranged between 1.000 and 1.322. Hence, the absence of collinearity within the model was established.

**Table 5:** Collinearity statistics (inner-VIF values).

	Inno	SE	SEG	VC
Inno			1.322	1.322
SE	1.000		1.322	1.322
SEG				
VC				

*N* = 343. SE, social entrepreneurship; inno, innovation; SEG, sustainable economic growth; VC, value creation.

### Structural Model

**Figure 3:**
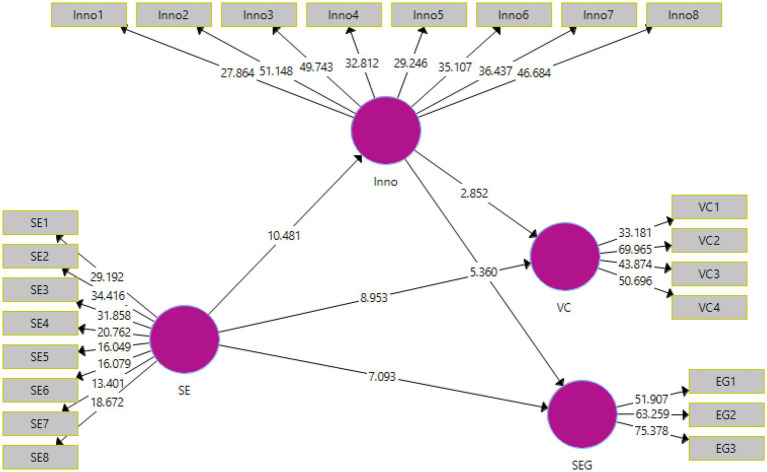
Structural model bootstrapping.

The output of the structural model can be viewed in Figure 3. The structural model depicts the values of t-statistics. The acceptance and rejection of the proposed hypotheses were determined by the PLS-SEM bootstrapping technique that was undertaken at 95% confidence interval.

The analysis of the direct and indirect effects can be viewed in Tables 6, 7. The acceptance or rejection of the proposed hypotheses was based on the t-statistic and *p*-values. The desirable t-statistic values should be above 1.96 (Johnson, 2019). Whereas, the *p*-value or the significance value should be below 0.05 (Di Leo and Sardanelli, 2020). Furthermore, the effect sizes were also recorded through the *f*-values. The effect sizes indicate the overall model strength. As per Meng and Bari (2019), the model strength is strong if the effect size is close to 1 and weak if it is close to 0.

**Table 6:** Direct effects of the variable.

Paths	H	O	M	SD	*t*-statistics	Effect sizes (f^2^)	*p*-values	Results
SE ➔ SEG	H1	0.438	0.434	0.062	7.093	0.256	0.000***	Accepted
SE ➔ VC	H2	0.547	0.547	0.061	8.953	0.397	0.000***	Accepted
SE ➔ Inno	H3	0.493	0.495	0.047	10.481	0.322	0.000***	Accepted
Inno ➔ SEG	H4	0.321	0.323	0.060	5.360	0.138	0.000***	Accepted
Inno ➔ VC	H5	0.180	0.181	0.063	2.852	0.043	0.005**	Accepted

*N* = 343. ***p < 0.001, **p < 0.005, SRMR = 0.017, NFI = 0.700. H, hypothesis; O, original sample; M, sample mean; SD, standard deviation; SE, social entrepreneurship; inno, innovation; SEG, sustainable economic growth; VC, value creation.

**Table 7:** Indirect effects of the variable.

Paths	H	O	M	SD	*t*-statistics	*p*	Results
SE ➔ Inno ➔ SEG	H6	0.158	0.159	0.031	5.123	0.000***	Accepted
SE ➔ Inno ➔ VC	H7	0.089	0.089	0.031	2.853	0.005**	Accepted

*N* = 343. ***p < 0.001, **p < 0.005. H, hypothesis; O, original sample; M, sample mean; SD, standard deviation; SE, social entrepreneurship; inno, innovation; SEG, sustainable economic growth; VC, value creation.

The analyses of the five direct hypotheses, i.e., H1, H2, H3, H4, and H5, are shown in Table 6. H1 predicted that social entrepreneurship (SE) had an effect on sustainable economic growth (SEG). The t-statistic and *p*-values are 7.093 and 0.000, respectively, which indicate the significance of the results. Therefore, H1 has been accepted. The effect size of 0.256 indicates weak model strength. H2 proposed that SE had an effect on value creation (VC). The t-statistic and *p*-values were 8.953 and 0.000, respectively, and therefore, H2 was also accepted. The effect size of 0.397 indicated weak to moderate model strength. H3 stated that SE had an effect on innovation. The t-statistic and *p*-values are 10.481 and 0.000, respectively, and therefore, H3 was also accepted. The effect size was recorded at 0.322 indicating weak model strength. H4 predicted that innovation had an effect on sustainable economic growth (SEG). The t-statistic and *p*-values are 5.360 and 0.000, respectively, and thus, H4 was accepted. The effect size was 0.138 indicating weak model strength. H5 proposed that innovation had an effect on VC. The t-statistic and *p*-values were 2.852 and 0.005, respectively, and therefore, H5 was also accepted.

The results of the indirect effects can be viewed in Table 7. H6 predicted that innovation mediated the relationship between social entrepreneurship (SE) and sustainable economic growth (SEG). The t-statistic and *p*-values were 5.123 and 0.000, respectively, and therefore, H6 was accepted. H7 proposed that innovation mediated the relationship between SE and value creation (VC). The t-statistic and *p*-values were 2.853 and 0.005, respectively, and therefore, H7 was also accepted.

## Discussion

The existing social and economics literature had a few gaps which have been addressed in the current study by examining the role of social entrepreneurship on sustainable economic growth and value creation. To carry out the research, the data were acquired from tour operators in China. The study also examined the indirect or mediating role of innovation in the relationship between social entrepreneurship and sustainable economic growth. The present investigation also analyzed the relationship between social entrepreneurship and value creation *via* innovation.

The intensive review of literature showcases the significant participation of psychology and psychologists in the field of entrepreneurship and social entrepreneurship. There is a potential for the psychologists in engaging with entrepreneurship and instigate novel topics in the research that facilitates and strengthens the psychological approaches in theory, practice and methodology to better understand the facets of entrepreneurship (Gorgievski and Stephan, 2016). Entrepreneurship is a potential driver for economic growth, societal productive and personal well being. The behavior of entrepreneurs can be researched to better support the organizational hierarchy and environment along with highlighting the brings side of the policy making for the entrepreneurs like job creation, innovation, poverty uplift, environmental sustainability and individual growth (Segal et al., 2005). Similarly, this would generate valuable understanding for broader behavioral research, such as how to cope the uncertainty in current pandemic affected trends in the world, how to increase flexibility of the work, responsibility which all are exhibited by the entrepreneurs. The first hypothesis (H1) of this study posited that social entrepreneurship has an effect on sustainable economic growth. This hypothesis was accepted. These results are harmonious with the findings of Palacios-Marqués et al. (2019) who opined that social entrepreneurial activities have been devised to reduce social problems; as a result, it boosts sustainable economic growth. Social entrepreneurs are responsible for developing strategies to mitigate social issues, which helps to accelerate sustainable economic growth.

The second hypothesis (H2) of this study posited that social entrepreneurship has an effect on value creation. This hypothesis was accepted. The results are in synchrony with the results of the study conducted by Yahchouchy and Dzenopoljac (2022a) which explained that social entrepreneurship is a phenomenon that boosts value creation because it aims to bring positive change to society and people. The reason is the capability of social entrepreneurs to carry out effective entrepreneurial activities to increase value creation. The third hypothesis (H3) of this study posited that social entrepreneurship has an effect on innovation. This hypothesis was accepted. Similar findings were obtained by Fridhi (2021) who asserted that social entrepreneurial activities are carried out for increasing the level of innovation in society to improve the overall living standard of the people. Social entrepreneurs use their skills to develop innovative activities to reduce social problems, thus social entrepreneurship and innovation are closely linked. The fourth hypothesis (H4) of this study posited that innovation has an effect on sustainable economic growth. This hypothesis was also accepted. Visvizi et al. (2018) also found that social entrepreneurship and innovation play an important role in boosting economic growth and development in the country. The reason is that innovation is a key for countries to have sustainable economic growth. The fifth hypothesis (H5) of this study posited that innovation has an effect on value creation. This hypothesis was also accepted. Similar findings were obtained by Adro and Fernandes (2021) who claimed that value creation originates from complementarity, novelty, and efficiency, and these three aspects are deeply rooted in innovation.

The results of the mediating role of innovation showed that innovation mediates the relationship between social entrepreneurship and sustainable economic growth. Thus, the sixth hypothesis (H6) was also accepted which posited that innovation has a mediating role in the relationship between social entrepreneurship and sustainable economic growth. These results are in harmony with the findings of Douglas and Prentice (2019) who argued that innovation is a critical indicator that impacts sustainable economic growth for improving living standards and social entrepreneurs are responsible for bringing this innovation. The innovative skills of social entrepreneurship enable innovation in society and this innovation leads to higher sustainable economic growth. The study also found that innovation has a mediating role between social entrepreneurship and value creation. Thus, the seventh hypothesis (H7) was accepted. Similar results were obtained by Méndez-Picazo et al. (2021) who exclaimed that the development of new products, innovation, and search for new opportunities positively impact economic growth which consequently influences value creation. Innovation is a power factor that is influenced by social entrepreneurs and helps to enhance value creation for the country or organization.

### Theoretical Implications, Practical Implication, Limitations and Future Direction, and Conclusion

#### Theoretical Implications

The current study incorporates some theoretical implications. First, the study aimed to examine the role of social entrepreneurship on sustainable economic growth and value creation. The findings greatly contribute to the literature on social economics and sustainability because few studies were conducted in this regard. Moreover, the study also improved the literature by analyzing the role played by innovation as a mediator. The reader could understand how innovation facilitates the relationship between social entrepreneurship and sustainable economic growth and between social entrepreneurship and value creation. Moreover, other researchers can enhance their knowledge with regard to the importance of social entrepreneurship and how it contributes to sustainable economic growth. The researchers can also understand the significant role played by innovation to enhance both sustainable economic growth and value creation.

#### Practical Implications

Taking the results of the study into account, it becomes imperative to provide practical guidelines for the policymakers and tour operators. Therefore, the present study proposed some practical implications. The results obtained depict that social entrepreneurship has a significant relationship with sustainable economic growth and value creation. This implies that policymakers must devise strategies and policies to encourage social entrepreneurship. This can be done by showing social entrepreneurs the kind of impact they can have on society. In this way, they would be motivated to serve society and work for public well-being. Another way that can be adopted by policymakers or government at the state level to boost social entrepreneurship is by giving them recognition. Moreover, social entrepreneurs must be encouraged by supporting the notion of social entrepreneurial activities. Social entrepreneurs work for society without expecting anything tangible in return; however, support from the government can boost their morale. Furthermore, the study found that innovation is a significant factor that facilitates the relationship between social entrepreneurship, sustainable economic growth, and value creation. Therefore, the management of tour operators must enhance the process of social entrepreneurship in order to accelerate innovation. Innovation acts as a catalyst that can boost value creation and economic development. Also, policy-makers and social entrepreneurs together can take advantage of the opportunities in terms of environmental aspects, economic aspects, and social aspects so that they can bring prosperity to society.

## Limitations and Recommendations

Like other studies, this study also includes a few limitations. First, this study has been conducted in China, therefore, future researchers can conduct the study in other Asian or western countries. The small sample size was another limitation of the study, and thus, a larger sample can be taken in future studies for data generalization. To carry out this study, the data were obtained from tour operators, so other industries or companies can also be considered in the future. This would enhance the understanding of the framework in other contexts as well. Another limitation of the study was the study design. The study was cross-sectional as the data were taken at one point in time. Future studies can use longitudinal data to examine how social entrepreneurship evolves. This study was quantitative; therefore, in the future, researchers can examine the same theoretical framework using qualitative data. Social entrepreneurship is a subjective phenomenon, so obtaining qualitative data would provide deep insights into the subject matter. Future studies can add new mediators or moderators to the current framework, for example, social change can be used as a mediator and good governance can be used as a moderator.

## Conclusion

Social entrepreneurship is a phenomenon that affects the economic growth of the country. Higher economic growth leads to a higher rate of employment and better living standards for the people in the society. To understand the impact of social entrepreneurship, this study examined the role of social entrepreneurship on sustainable economic growth and value creation among tour operators in China. The study also determined the mediating effect of innovation in the relationship between social entrepreneurship and sustainable economic growth and between social entrepreneurship and value creation. This investigation revealed that social entrepreneurship has an impact on sustainable economic growth, value creation, and innovation. The results obtained also showed that innovation impacts sustainable economic growth and value creation. The study found that innovation mediates the relationship between social entrepreneurship and sustainable economic growth and between social entrepreneurship and value creation among tour operators in China.

## Data Availability Statement

The original contributions presented in the study are included in the article/supplementary material, further inquiries can be directed to the corresponding author.

## Ethics Statement

The studies involving human participants were reviewed and approved Shandong University of Arts, China. The patients/participants provided their written informed consent to participate in this study. The study was conducted in accordance with the Declaration of Helsinki.

## Author Contributions

WW conceptualized the concept, collected the data, analyzed the data, wrote the draft, and approved the submitted version.

## Conflict of Interest

The author declares that the research was conducted in the absence of any commercial or financial relationships that could be construed as a potential conflict of interest.

## Publisher’s Note

All claims expressed in this article are solely those of the authors and do not necessarily represent those of their affiliated organizations, or those of the publisher, the editors and the reviewers. Any product that may be evaluated in this article, or claim that may be made by its manufacturer, is not guaranteed or endorsed by the publisher.
